# Supplementary material for: Beneficial Exercises for Cancer-Related Fatigue among Women with Breast Cancer: A Systematic Review and Network Meta-Analysis
Source: Cancers (Basel). 2022 Dec 27;15(1):151. doi: 10.3390/cancers15010151 (PMC9817866; doi:10.3390/cancers15010151)
Supplement: Supplementary file 1 [file cancers-15-00151-s001.zip › Supplementary figures.pdf]

Figure S1. PRISMA flowchart for selected studies

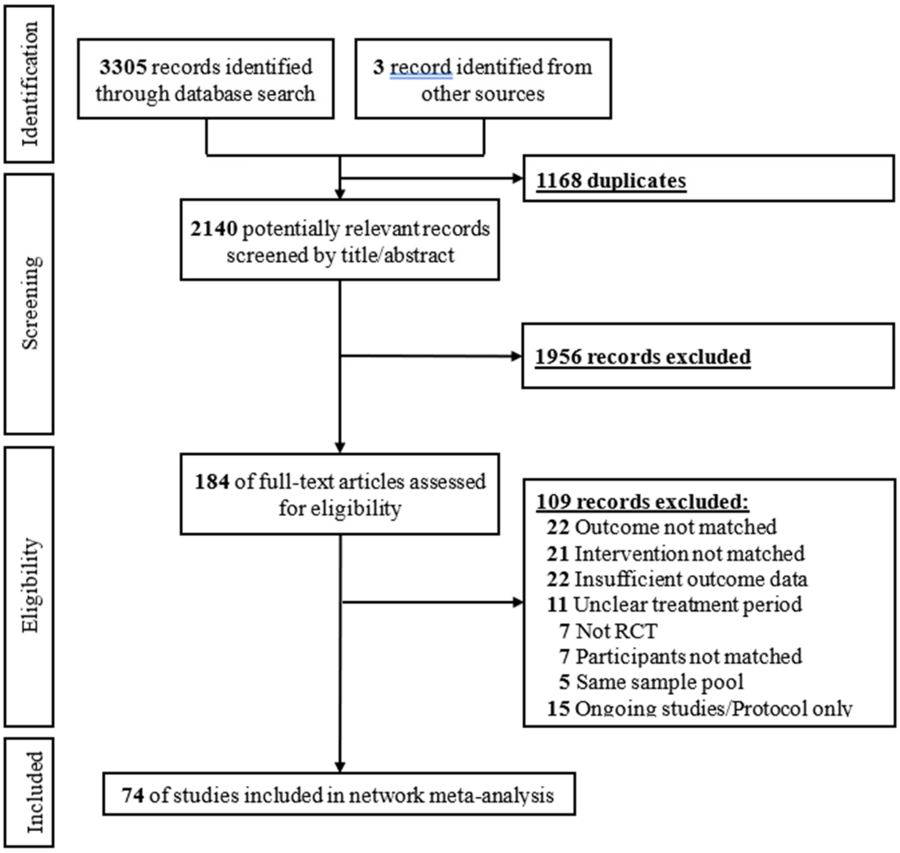

Figure S2. RoB of included selected studies

	Study ID	D1	D2	D3	D4	D5	Overall
**Inter-treatment**	Al-Majid 2015	!	!	+	!	!	!
	Battaglini 2006	-	!	!	-	-	-
	Bolam 2019	+	!	+	!	+	!
	Campbell 2005	!	!	!	!	-	-
	Cešeiko 2019	!	+	+	+	+	+
	Chandwani 2010	!	!	!	!	!	!
	Chandwani 2014	!	!	!	!	+	!
	Chaoul 2018	!	-	!	!	!	-
	Chen 2013	!	!	!	!	+	!
	Cornette 2016	!	-	!	!	!	-
	Courneya 2007	+	!	+	!	!	!
	Danhauer 2015	!	+	+	+	!	!
	Gokal 2016	!	!	+	!	!	!
	Hu 2013	!	!	!	+	+	!
	Huang 2019	+	+	+	+	+	+
	Husebø 2014	+	!	+	!	+	!
	Hwang 2008	!	!	!	!	-	-
	Jong 2018	!	!	-	!	!	-
	Kirkham 2020	!	!	!	!	+	!
	Lee 2021	+	+	+	+	+	+
	Lötzke 2016	-	-	!	!	+	-
	Mijwel 2018	+	-	-	!	!	-
	Mock 2005	+	!	+	!	!	!
	Møller 2020	!	!	+	!	+	!
	Mostafaei 2021	+	!	+	!	+	!
	Mutrie 2007	+	!	!	!	+	!
	Naraphong 2015	!	!	+	!	!	!
	Schmidt 2015a	!	+	+	+	!	!
	Schmidt 2015b	+	!	!	!	!	!
	Steindorf 2014	+	+	+	+	+	+
	Taso 2014	!	!	+	!	+	!
	van Waart 2015	!	!	!	!	+	!
	VanderWalde 2020	+	+	+	+	+	+
	Vadiraja 2009	+	+	-	+	!	-
	Wang 2011	!	!	!	!	+	!
	Wang 2014	-	!	!	!	!	-
**Post-treatment**	Aydin 2021	+	+	+	+	+	+
	Baglia 2019	+	+	+	+	+	+
	Banasik 2011	!	!	!	!	-	-
	Bower 2005	+	+	!	+	!	!
	Carson 2009	!	!	!	!	-	-
	Cohen 2021	+	!	+	!	+	!

\+ Low risk
! Some concerns
\- High risk

D1 Randomisation process
D2 Deviations from the intended interventions
D3 Missing outcome data
D4 Measurement of the outcome
D5 Selection of the reported result

**Study ID**	**D1**	**D2**	**D3**	**D4**	**D5**	**Overall**
Cramer 2015	+	!	+	!	+	!
Demello 2018	+	+	+	+	+	+
Dieli-Conwright 2018	+	+	+	+	+	+
Do 2014	-	!	-	!	!	-
Ergun 2013	!	!	!	!	!	!
Gal 2021	+	!	+	!	+	!
Hagstrom 2016	+	!	!	!	-	-
Jang 2021	!	!	!	!	+	!
Kiecolt-Glaser 2014	+	!	!	!	+	!
Kim 2020	!	!	+	!	+	!
Littman 2012	!	!	!	!	!	!
Loh 2014	+	!	!	!	!	!
Milne 2018	!	!	+	!	+	!
Moraes 2021	!	!	+	!	+	!
Natma 2015	+	!	+	!	-	-
Naumann 2012	!	!	!	!	-	-
Ochi 2021	+	+	+	+	+	+
Pagola 2020	!	!	+	+	+	!
Paulo 2019	!	!	+	+	+	!
Pinto 2003	!	!	-	!	-	-
Pinto 2008	!	!	-	!	!	-
Rogers 2014	+	!	+	!	!	!
Rogers 2017	+	!	+	!	+	!
Saarto 2012	+	!	!	!	+	!
Santagenello 2020	!	+	!	!	+	!
Schmidt 2017	+	!	!	!	!	!
Stan 2016	!	-	-	!	-	-
Taylor 2018	+	-	-	!	-	-
Winters-Stone 2012	+	!	!	+	!	!
Yagli 2015	!	+	+	!	-	-
Yağli 2015	!	!	!	!	-	-
Yuen 2007	!	!	!	!	-	-
