# Supplementary material for: Beneficial Exercises for Cancer-Related Fatigue among Women with Breast Cancer: A Systematic Review and Network Meta-Analysis
Source: Cancers (Basel). 2022 Dec 27;15(1):151. doi: 10.3390/cancers15010151 (PMC9817866; doi:10.3390/cancers15010151)
Supplement: Supplementary file 1 [file cancers-15-00151-s001.zip › Table S2 - Searching process.pdf]

S2 1 Searching strategies of four databases

Database	#	Searching Strategy	Results
1) Embase	1	(Mastectomy OR ((breast OR mamma*) NEAR/3 (cancer* OR neopla* OR adenocarcin* OR carcin* OR tumor* OR tumour* OR malignan* OR sarcoma* OR mass* OR DCIS OR ductal* OR infiltrat* OR intraductal* OR lobula* OR medullary))):ti,ab,kw,de	585528
	2	"breast tumor"/exp OR Mastectomy/exp	502000
	3	(Exercis* OR kinesio* OR sport* OR training* OR yoga OR "tai chi" OR "meditative movement" OR aerobic* OR danc* OR strength* OR walk* OR jog* OR bicycl* OR swimming OR pilates OR (physical NEAR/3 (activit* OR conditioning* OR effort* OR exertion* OR endurance* OR restraint* or movement*))):ti,ab,kw,de	1783392
	4	Exercise/exp OR kinesiotherapy/exp OR "physical activity"/exp OR sport/exp OR training/exp OR dancing/exp OR "muscle strength"/exp	791775
	5	(Fatigue* OR Tired* OR Lassitude OR Exhausti* OR weary OR weariness):ti,ab,kw,de	274920
	6	Fatigue/exp "Fatigue Severity Scale"/exp OR "Fatigue Impact Scale"/exp	1821
	7	(#1 OR #2) AND (#3 OR #4) AND (#5 OR #6)	1752
	8	#7 AND [embase]/lim	1601
	9	#8 AND ('crossover procedure':de OR 'double-blind procedure':de OR 'randomized controlled trial':de OR 'single-blind procedure':de OR (random* OR factorial* OR crossover* OR cross NEXT/1 over* OR placebo* OR doubl* NEAR/1 blind* OR singl* NEAR/1 blind* OR assign* OR allocat* OR volunteer*):de,ab,ti)	648
2) Medline	1	(Malignan* OR Mastectomy OR ((breast OR mamma*) ADJ4 (cancer* OR neopla* OR adenocarcin* OR carcin* OR tumor* OR tumour* OR malignan* OR sarcoma* OR mass* OR DCIS OR ductal* OR infiltrat* OR intraductal* OR lobula* OR medullary))).mp	871345
	2	exp "Breast Neoplasms"/ OR exp "Oncology nursing"/ OR exp Mastectomy/	278338
	3	(Exercis* OR kinesio* OR sport* OR training* OR "weight training" OR yoga OR "tai chi" OR "meditative movement" OR aerobic* OR danc* OR strength* OR walk* OR jog* OR bicycl* OR swimming OR pilates OR (physical ADJ4 (activit* OR conditioning* OR effort* OR exertion* OR endurance* OR restraint* or movement*))).mp	1331543
	4	exp Exercise/ OR exp "Exercise Therapy"/ OR exp Sports/ OR exp "Physical Exertion"/ OR exp "Exercise Movement	341836

		Techniques"/ OR exp "Physical Fitness"/ OR exp "Muscle Strength"/	
	5	(Fatigue* OR Tired* OR Lassitude OR Exhausti*).mp	130161
	6	Exp Fatigue/	27076
	7	(1 OR 2) AND (3 OR 4) AND (5 OR 6)	830
	8	7 and (Randomized controlled trial.pt. or controlled clinical trial.pt. or randomi*ed.ab. or placebo.ab. or drug therapy.fs. or randomly.ab. or trial.ab. or groups.ab. not (exp animals/ not humans.sh.))	466
3) CINHAL	1	Malignan* OR Mastectomy OR ((breast OR mamma*) N3 (cancer* OR neopla* OR adenocarcin* OR carcin* OR tumor* OR tumour* OR malignan* OR sarcoma* OR mass* OR DCIS OR ductal* OR infiltrat* OR intraductal* OR lobula* OR medullary))	135021
	2	MH ("Breast Neoplasms+" OR "Oncologic Nursing+" OR Mastectomy+)	84057
	3	Exercis* OR kinesio* OR sport* OR training* OR "weight training" OR yoga OR "tai chi" OR "meditative movement" OR aerobic* OR danc* OR strength* OR walk* OR jog* OR bicycl* OR swimming OR pilates OR (physical N3 (activit* OR conditioning* OR effort* OR exertion* OR endurance* OR restraint* or movement*))	476799
	4	MH (Exercise+ OR "Exercise Therapy+" OR Sports+ OR "Physical Exertion+" OR "Exercise Movement Techniques+" OR "Physical Fitness+" OR "Muscle Strength+")	162119
	5	Fatigue* OR Tired* OR Lassitude OR Exhausti*	44456
	6	MH (Fatigue+)	18675
	7	(s1 OR s2) AND (s3 OR s4) AND (s5 OR s6)	641
	8	s7 and ((MH "Clinical Trials+") or (PT Clinical trial) or (TX clinic* n1 trial*) or TX ((singl* n1 blind*) or (singl* n1 mask*)) or TX ((doubl* n1 blind*) or (doubl* n1 mask*)) or TX ((tripl* n1 blind*) or (tripl* n1 mask*)) or TX ((trebl* n1 blind*) or (trebl* n1 mask*)) or (TX randomi* control* trial*) or (MH "Random Assignment") or (TX random* allocat*) or (TX placebo*) or (MH "Placebos") or (MH "Quantitative Studies") or (TX allocat* random*))	420
4) CENTRAL	1	(Malignan* OR Mastectomy OR ((breast OR mamma*) NEAR/3 (cancer* OR neopla* OR adenocarcin* OR carcin* OR tumor* OR tumour* OR malignan* OR sarcoma* OR mass* OR DCIS OR ductal* OR infiltrat* OR intraductal* OR lobula* OR medullary))):ti,ab,kw	42798
	2	[mh "Breast Neoplasms"] OR [mh "Oncology nursing"] OR [mh Mastectomy]	11353
	3	(Exercis* OR kinesio* OR sport* OR training* OR "weight	143366

		training" OR yoga OR "tai chi" OR "'meditative movement" OR aerobic* OR danc* OR strength* OR walk* OR jog* OR bicycl* OR swimming OR pilates OR (physical NEAR/3 (activit* OR conditioning* OR effort* OR exertion* OR endurance* OR restraint* or movement*))):ti,ab,kw	
	4	[mh Exercise] OR [mh "Exercise Therapy"] OR [mh Sports] OR [mh "Physical Exertion"] OR [mh "Exercise Movement Techniques"] OR [mh "Physical Fitness"] OR [mh "Muscle Strength"]	35445
	5	(Fatigue* OR Tired* OR Lassitude OR Exhausti*):ti,ab,kw	25914
	6	[mh Fatigue]	2991
	7	(#1 OR #2) AND (#3 OR #4) AND (#5 OR #6)	546
